# Inorganic carbon distribution and CO_2_ fluxes in a large European estuary (Tagus, Portugal)

**DOI:** 10.1038/s41598-017-06758-z

**Published:** 2017-08-07

**Authors:** A. P. Oliveira, G. Cabeçadas, M. D. Mateus

**Affiliations:** 1 Instituto Português do Mar e da Atmosfera (IPMA), I. P., Av. Brasília, 1449-006 Lisbon, Portugal; 2 Instituto Superior de Educação e Ciências de Lisboa (ISEC Lisboa), Lisbon, Portugal; 3 MARETEC, Instituto Superior Técnico, Universidade de Lisboa, Av. Rovisco Pais, 1049-001 Lisboa, Portugal

## Abstract

Ten field cruises were carried out in Tagus estuary from 1999 to 2007 to study the dynamics of the inorganic carbon system. Dissolved inorganic carbon (DIC) and total alkalinity (TA) increased with salinity. DIC and TA were generally conservative in the estuarine mixing zone (salinity > 10), while a complex distribution pattern was observed at the upper estuary. DIC values peaked 1786.9 ± 155.8 µmol kg^−1^ at that segment. Estimated annual mean fluxes of DIC were 0.27 Tg C yr^−1^ from the river to the estuary, and 0.37 Tg C yr^−1^ from here to the coastal area. The Tagus estuary was always CO_2_ supersaturated, with partial pressure of CO_2_ (*p*CO_2_) reaching 9160 µatm in the upper estuary. An average emission of 0.11 Tg C yr^−1^ was estimated from the estuary to the atmosphere, corresponding to 23% of exported DIC. Only 8% of the riverine DIC was ventilated. The non-conservative behaviour of CO_2_ parameters in the estuary segment under freshwater influence was attributed to alternations in the relevance of riverine/terrestrial runoff, photosynthesis, aerobic respiration, organic matter mineralization and CaCO_3_ precipitation/dissolution.

## Introduction

Estuaries rank among the most productive and dynamic aquatic ecosystems^1^. They are frequently characterized by strong physical-chemical gradients, enhanced biological activity and intense sediment dynamics. Nowadays it is unequivocally accepted that inner estuaries act as sources of CO_2_ to the atmosphere. A recent study^2^ reports that estuaries emit 20.8 mol C m^−2^ yr^−1^ to the atmosphere, mainly due to their heterotrophic metabolic status, sustained by terrestrial/riverine organic carbon inputs, and also by waste water in populated areas. A fraction of this carbon is exported to the nearby coastal areas mostly as organic particles, but also to the atmosphere in the form of CO_2_ emissions. Hence, inner estuaries are effective sieves for terrestrial/riverine inputs and provide a by-pass of carbon towards the atmosphere^3^.

The flux and/or residence of carbon in each of these compartments depend on the characteristics of the estuary, as well as on the season, and even time of day. For example, approximately 60% of the respiratory CO_2_ in Scheldt estuary is released to the atmosphere, 26% transferred to the sediment, and only 14% remains in the water column^1^. Moreover, spatial variability plays an important role due to the hydrodynamic and geomorphological complexity of these littoral zones. Thus, fluxes, sources and mechanisms of CO_2_ transport and transformation are among the most important current issues in marine and freshwater geochemistry.

A compilation^4^ of available data on water-air CO_2_ fluxes in inner estuaries shows that the west European inner estuaries have been extensively studied, accounting for 47% of the total results presented. So far, inter-annual and decadal variability of water-air CO_2_ fluxes is still undocumented in some estuarine environments. Few studies have been undertaken involving CO_2_ fluxes variability in Portugal, a country at the eastern boundary of the Subtropical North Atlantic. Data of water-air CO_2_ fluxes are available for just three Portuguese estuarine systems: the Aveiro coastal Lagoon, the Douro and Sado estuaries^5^. The Douro, Tagus and Sado inner estuaries and their adjacent coastal waters behave as sources of CO_2_ to the atmosphere^6^, with fluxes ranging from 31 to 76 mol C m^−2^ d^−1^, which corresponds to an additional source of 0.1% to the CO_2_ emissions by the near-shore ecosystems^4^. However, the estuarine inorganic carbon dynamics has never been assessed.

**Figure 1:**
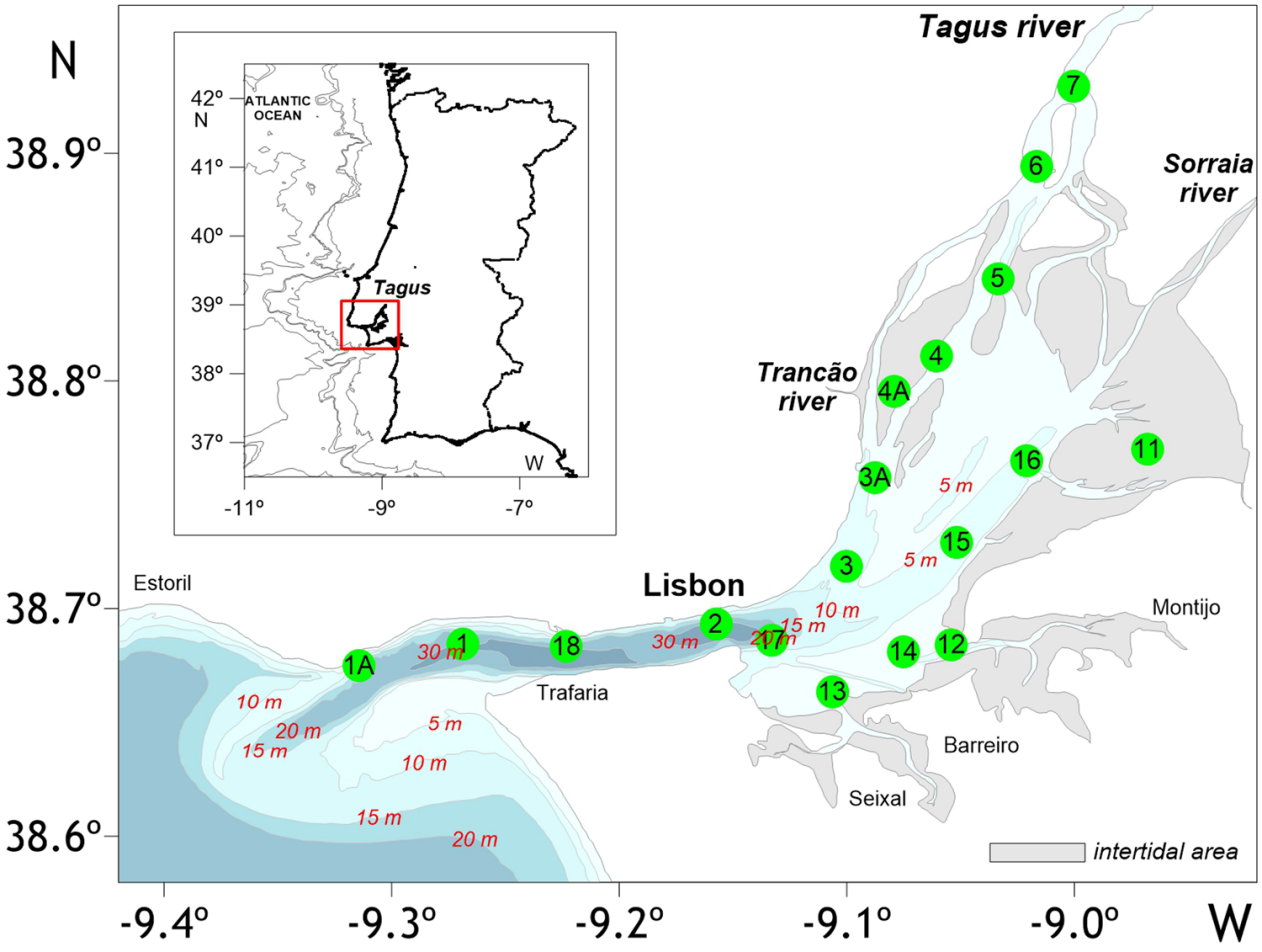
Map of Tagus estuary with the location of the sampling stations. Figure generated using the software Surfer Version 12.8.1009 (http://www.goldensoftware.com/products/surfer), a surface mapping system from Golden Software, LLC. Coastline and bathymetry were created based on Google “Map data: Google, Earth” (https://www.google.pt/maps/) and on data SIO, NOAA, U.S. Navy, NGA, GEBCO version 20141103 (http://www.gebco.net).

This paper intends to partially fulfil this gap by focusing on the CO_2_ dynamics along Tagus estuary (Fig. 1), Portugal, one of the largest western European estuaries in surface area. Spatial and temporal CO_2_ variability is addressed in this estuary based on information obtained from 10 surveys carried out from 1999 to 2007. This work also intends to identify and study the dominant factors/mechanisms influencing the inorganic carbon system. In addition, some estimates are advanced on the water-air CO_2_ fluxes over the estuary.

## Results

### General water properties

**Figure 2:**
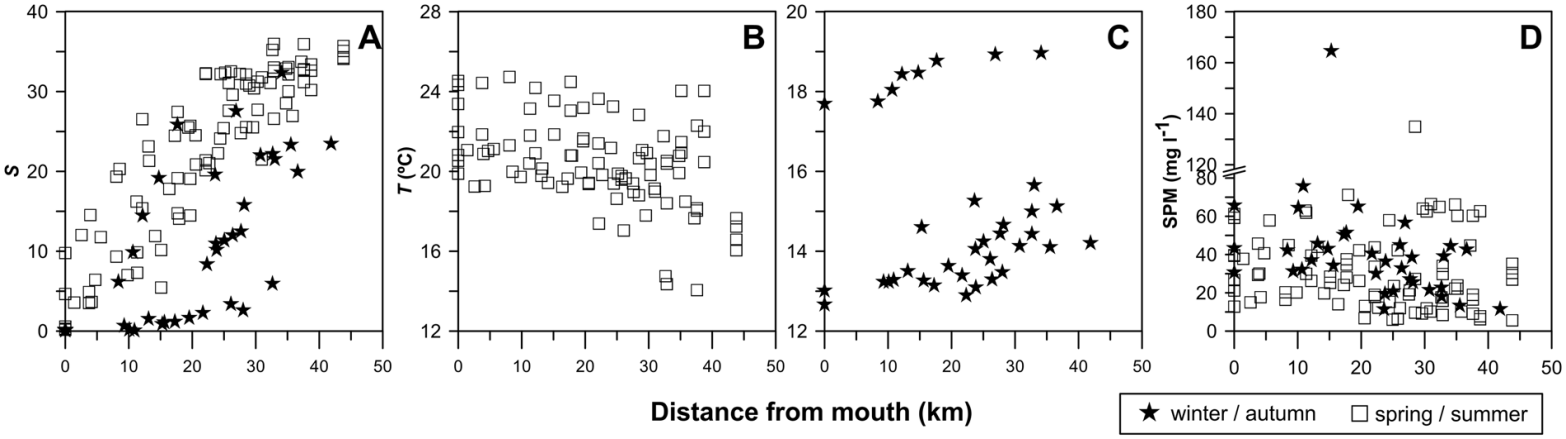
Longitudinal distributions of (**A**) salinity (*S*), (**B**) temperature (*T*) in spring/summer, (**C**) temperature (*T*) in winter/autumn and (**D**) suspended particulate matter (SPM), during the 10 surveys carried out along Tagus estuary. Riverine end-member: sampling station with lowest salinity; Marine end-member: sampling station nearest the ocean.

**Figure 3:**
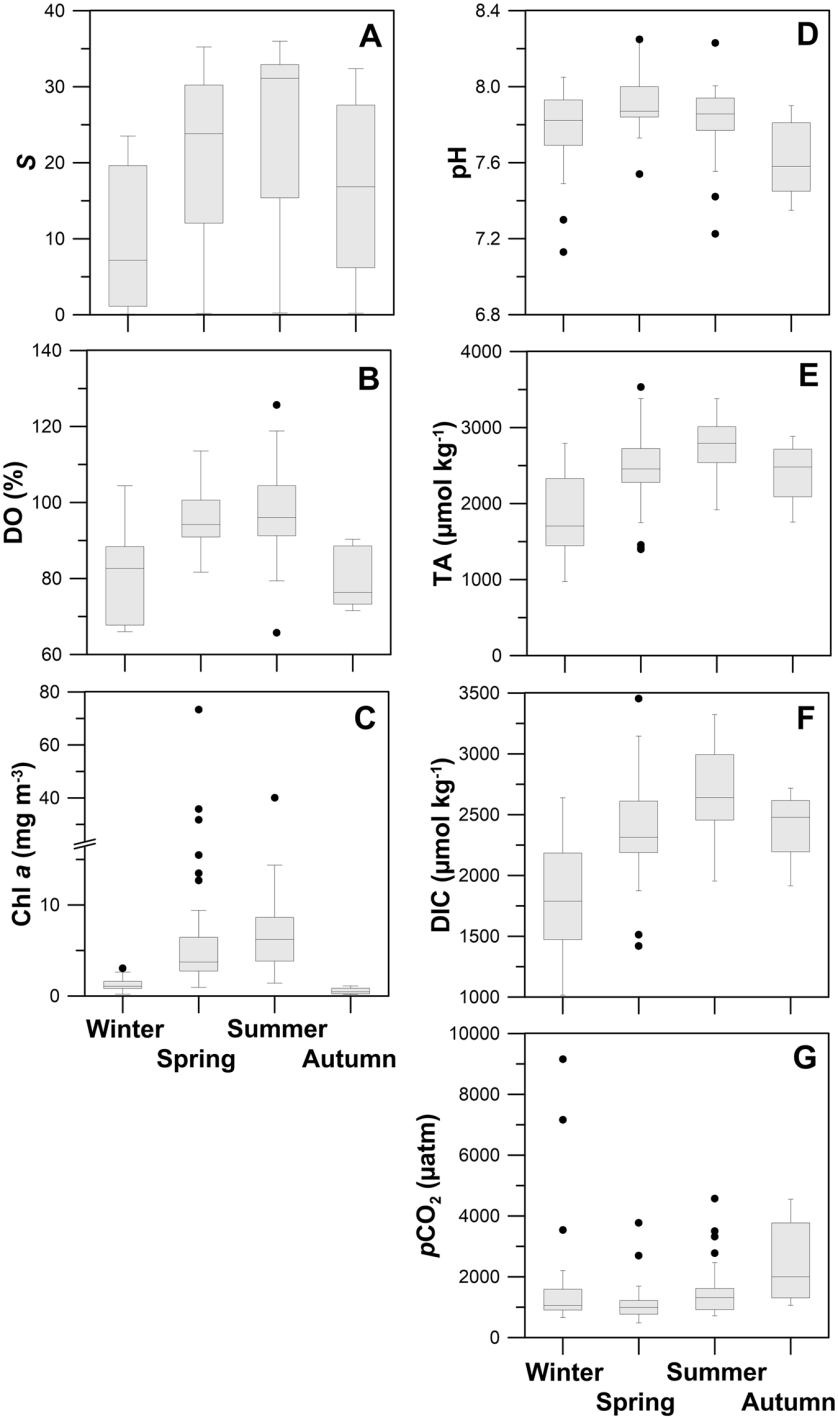
Box-whisker plot of (**A**) salinity (*S*), (**B**) dissolved oxygen (DO), (**C**) chlorophyll *a* (Chl *a*), (**D**) pH, (**E**) total alkalinity (TA), (**F**) dissolved inorganic carbon (DIC) and (**G**) CO_2_ partial pressure (*p*CO_2_) in Tagus estuary during 1999–2007. Median values are represented by line inside boxes, 25th to 75th percentiles are denoted by box edges, 10th to 90th percentiles are depicted by the error bars, and outliers are indicated by circles.

The range of values for physical, chemical and biological variables from 1999 to 2007 is shown in Table 1. Overall, salinity increased downstream (Fig. 2A) with lower values in winter/autumn (Fig. 3A). The environmental conditions in the estuary end-members indicate that salinity kept reasonably stable at the marine influenced area in most samples (Table 1), the exception being winter seasons and May 2000. In March 2001 the river discharge peaked 1861 m^3^ s^−1^ and salinity did not exceed 12.5 in the estuary. A decrease in temperature from the upper to the lower estuary was observed during spring and summer (Fig. 2B). This was in contrast with winter and autumn, when an increase down the estuary was seen (Fig. 2C). While the amplitude of temperature was 12 °C in the river end-member, only 5 °C amplitude was recorded at the marine influenced area.

**Table 1:** Range of physical-chemical and biological properties of surface waters in Tagus estuary for each sampling date. Freshwater flow (Q), salinity (*S*), temperature (*T*), suspended particulate matter (SPM), chlorophyll *a* (Chl *a*), dissolved oxygen (DO), total alkalinity (TA), dissolved inorganic carbon (DIC) and CO_2_ partial pressure (*p*CO_2_).

Season	Sampling dates	Q ^a^ (m^3^ s^−1^)	*S*	*T* (°C)	SPM (mg l^−1^)	Chl *a* (mg m^−1^)	DO (%)	pH	TA (µmol kg^−1^)	DIC (µmol kg^−1^)	*p*CO_2_ (µatm)
Summer	September 1999^b^	33	4.66–36.00	14.1–22.0	16.4–39.6	1.5–14.4	79–106	7.23–7.91	2307–3381	2409–3323	922–4575
Spring	May 2000	376	0.17–26.96	17.8–20.8	6.1–71.3	1.0–13.5	82–103	7.82–7.88	1401–3535	1421–3455	906–1592
Winter	March 2001	1861	0.09–12.50	13.0–14.6	19.6–164.7	0.7–3.1	66–104	7.63–7.92	977–1834	1016–1816	873–1998
Summer	July 2001^b^	130	0.24–35.10	16.1–24.7	7.8–42.3	3.2–13.0	93–126	7.91–8.01	1918–2827	1954–2666	714–1326
Summer	June 2002^b^	81	0.31–34.27	17.7–24.3	28.6–135.0	1.4–40.1	87–104	7.70–8.23	2573–3330	2587–3270	1012–2779
Spring	May 2003^b^	233	0.20–34.12	16.6–23.4	5.7–62.0	1.6–31.8	85–107	7.54–7.94	1751–2784	1875–2672	851–3778
Winter	February 2004	264	0.18–23.51	12.7–15.7	11.5–64.5	0.2–2.6	78–91	7.13–8.05	1447–2792	1476–2639	662–9160
Spring	May 2006^b^	125	0.58–33.76	17.7–21.8	37.9–116.3	1.0–73.4	89–114	7.77–8.25	1418–3382	1422–3145	487–2704
Autumn	November 2006	768	0.21–32.39	17.7–19.0	32.3–56.8	0.2–1.1	72–90	7.35–7.90	1757–2884	1915–2717	1058–4552
Spring	May 2007^b^	86	9.81–35.23	14.8–20.9	12.0–45.0	1.4–9.4	82–101	7.89–8.05	2371–2528	2269–2366	620–970

^a^Tagus river flow, taken from Almourol station (the most downstream hydrological station, ~85 km upstream the estuary mouth). On-the-spot water discharge data was not available. Values were obtained from the Portuguese Environment Agency (APA, I.P.) accessed in a public database (http://snirh.apambiente.pt/)

^b^Upwelling was present at the adjacent coastal shelf.

Due to hydrodynamic conditions imposed by the tidal regime (tidal amplitude of 1.5 to 4.0 m) and the water discharges (Tagus discharges from 33 to 1861 m^3^ s^−1^; Table 1), significant amounts of suspended matter spread all over the estuary (Fig. 2D). In general the amount of SPM in the estuary was low to moderate, rarely exceeding 80 mg l^−1^ (Fig. 2D; Table 1).

Concerning oxygenation conditions, Tagus waters were always well oxygenated, in general displaying saturation levels above 70% (Table 1), with an increase from winter to spring/summer (Fig. 3B). Concentration of phytoplankton biomass (Chl *a*) reached a maximum of 73.4 mg m^−3^ in May 2006, and hardly attained 3 mg m^−3^ (Fig. 3C; Table 1) in the rest of the periods (winter/autumn). An increase of Chl *a* towards the fluvial section was generally observed.

### Variations of inorganic carbon system parameters

**Figure 4:**
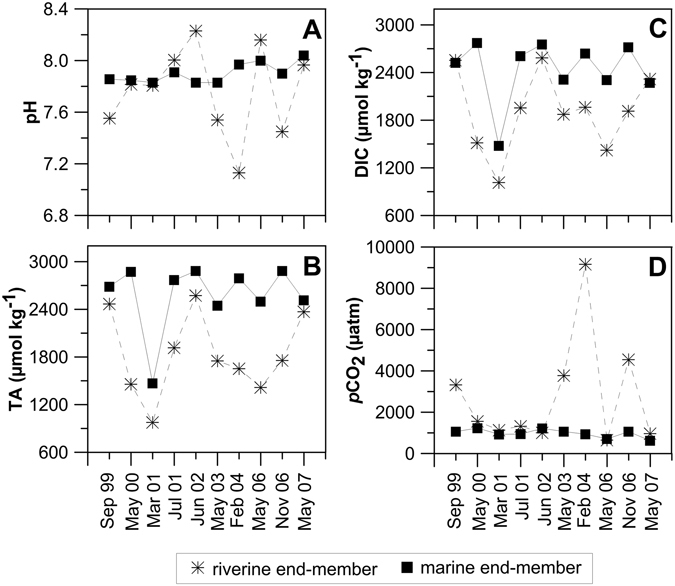
Riverine (✳) and marine (■) end-members values of (**A**) pH, (**B**) total alkalinity (TA), (**C**) dissolved inorganic carbon (DIC) and (**D**) CO_2_ partial pressure (*p*CO_2_) throughout to the 10 surveys undertaken in Tagus estuary.

**Figure 5:**
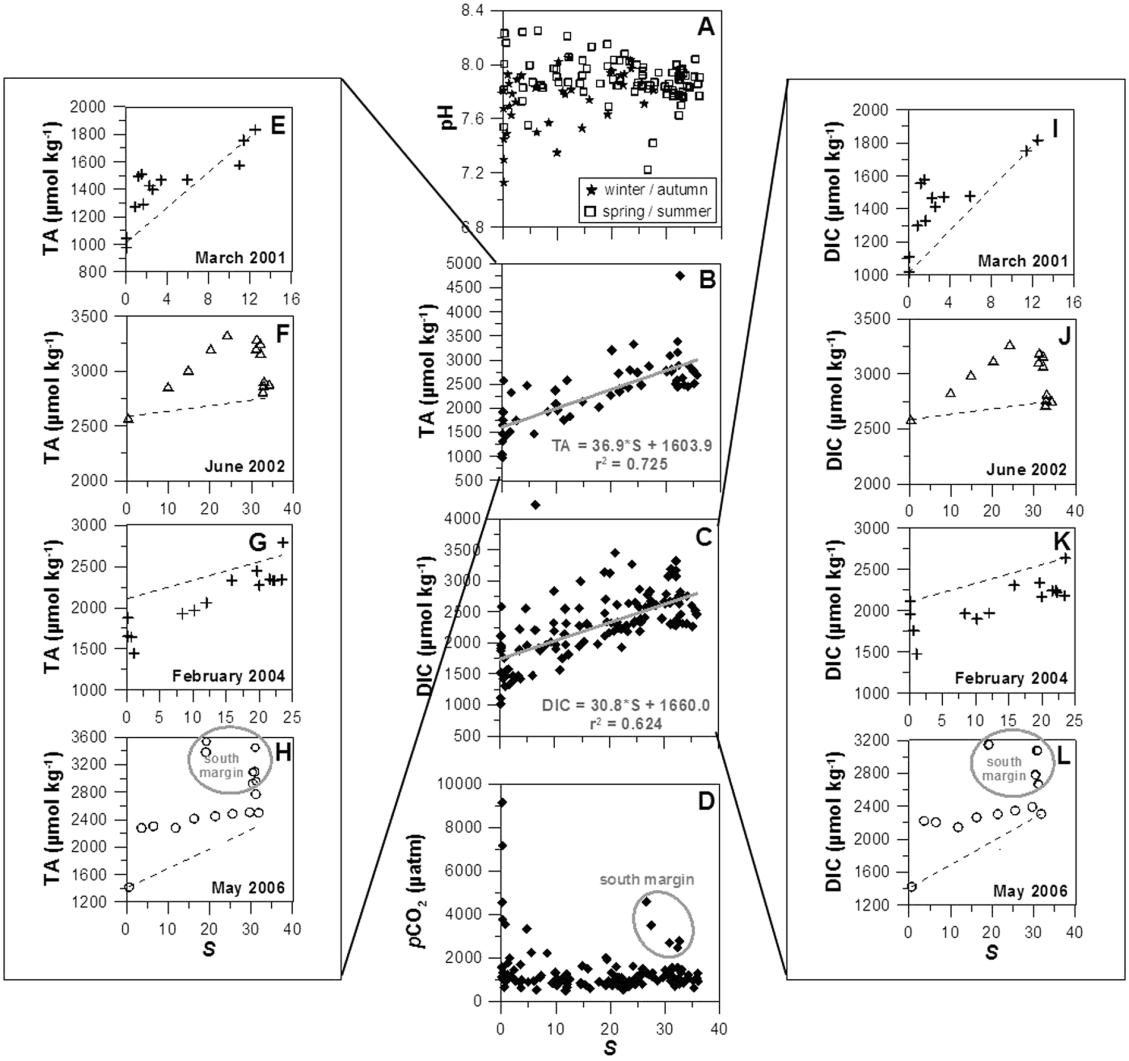
Mixing curves for (**A**) pH, (**B**) total alkalinity (TA), (**C**) dissolved inorganic carbon (DIC) and (**D**) CO_2_ partial pressure (*p*CO_2_) for all 10 surveys data undertaken in Tagus estuary. TA anomalies in (**E**) March 2001, (**F**) June 2002, (**G**) February 2004 and (**H**) May 2006. DIC anomalies in (**I**) March 2001, (**J**) June 2002, (**K**) February 2004 and (**L**) May 2006.

pH values in the marine section were rather constant (amplitude 0.21; Fig. 4A), while the riverine end-member pH varied significantly (amplitude 1.10; Fig. 4A). Seasonally, pH showed scattered patterns and variations were not the same for all sampling periods (Fig. 5A): in autumn/winter more acidic features were noticed upstream (7.13–7.68) and increased downstream (7.83–8.03); in spring/summer, most of the time, more basic values were present upstream than downstream.

TA and DIC values were always lower in the riverine section than in the marine area, ranging between 977 µmol kg^−1^ in winter and 2587 µmol kg^−1^ in summer. These parameters displayed less irregular values in the marine influenced area and varied, respectively, from 1469 µmol kg^−1^ in winter to 2885 µmol kg^−1^ in summer (Fig. 4B,C). A negative correlation was found between riverine TA and average freshwater discharges (r^2^ = 0.482, *p* < 0.05). TA and DIC increased from winter to spring/summer (Fig. 3E,F), with values varying from 1401 to 3535 µmol kg^−1^ during the productive period and from 977 to 2884 µmol kg^−1^ during the non-productive period (Table 1).

*p*CO_2_ values were higher in the riverine section, reaching as high as 9160 µatm in February 2004 (Fig. 4D; Table 1), a value ~24 times higher than *p*CO_2_ of atmospheric equilibrium (383.9 µatm). Seasonally average values of *p*CO_2_ remain rather stabilized from winter to spring/summer and increased in autumn (Fig. 3G). Values of *p*CO_2_ dropped rapidly in the upper estuarine zone (Fig. 5D) at salinities below 10. *p*CO_2_ values as high as 9160 µatm (Table 1) were accompanied by low pH (7.13) at salinities below 5. Downstream, *p*CO_2_ in general dropped to values of 620 µatm at the estuary mouth (Fig. 5D).

### CO_2_ evasion pattern

Table 2 shows CO_2_ gas transfer velocities at the water-air interface, proposed by different authors and calculated for Tagus estuary, as well as wind speed and tidal current for each season. Daily wind velocity was random and variable, oscillating between 1.7 m s^−1^ and 3.9 m s^−1^. The maximum tidal current was lower in summer (58 cm s^−1^) and reached 122 cm s^−1^ in autumn depending on the tidal regime, river discharges and bathymetry of the estuary. Two of the algorithms used to estimate gas transfer velocity (*k*), namely *k*_B04_^7^ and *k*_A09_^8^, gave the highest values when compared with other algorithms. This was an expected outcome since both approaches are based on the same technique (floating dome) and took into account the water current velocity. The *k*_OD58_^9^ algorithm led to minimum *k* values, since it considers water current alone. However, maximum values of *k* were obtained in spring for all the parameterizations associated with maximum wind speed conditions (Table 2).

**Table 2:** Tagus estuary seasonal values for daily average wind speed, maximum tidal current speed, *p*CO_2_ gradient (Δ*p*CO_2_), CO_2_ gas transfer velocity (*k*) given by the parameterizations of Carini *et al*.^42^ (*k*_C96_), Raymond and Cole^43^ (*k*_RC01_), Abril *et al*.^8^ (*k*_RC01_), Borges *et al*.^7^ (*k*_B04_) and O**’**Connor and Dobbins^9^ (*k*_OD58_), water current contribution, water-air CO_2_ fluxes and CO_2_ emission.

	winter	spring	summer	autumn
Wind speed (m s^−1^)	3.7 ± 1.6	3.9 ± 1.8	3.3 ± 1.3	1.7 ± 0.7
Maximum tidal current speed (cm s^−1^)	103	80	58	122
Δ*p*CO_2_ (µatm)	1337.1 ± 1930.8	674.2 ± 532.9	1147.4 ± 872.6	1908.6 ± 1277.8
*k*_C96_ (cm h^−1^)	5.0 ± 2.0	7.1 ± 3.7	4.9 ± 1.8	3.3 ± 1.4
*k*_RC01_ (cm h^−1^)	4.8 ± 1.7	8.1 ± 6.3	4.6 ± 1.3	3.3 ± 0.8
*k*_B04_ (cm h^−1^)	11.9 ± 3.6	14.9 ± 4.8	11.6 ± 2.7	9.9 ± 3.7
*k*_A09_ (cm h^−1^)	10.3 ± 3.6	14.1 ± 6.6	10.4 ± 3.2	7.3 ± 2.4
*k*_OD58_ (cm h^−1^)	4.6 ± 1.4	4.6 ± 1.5	4.0 ± 1.6	4.6 ± 2.9
Water current contribution (%)^a^	48	39	45	59
Water-air CO_2_ flux (mmol C m^−2^ d^−1^)^b^	109.9 ± 83.9	80.4 ± 87.7	86.9 ± 64.6	133.9 ± 89.3
CO_2_ emission (10^6^ mol C d^−1^)^c^	29.0	21.2	23.0	35.3

^a^calculated as the ratio *k*_OD58_/(*k*_OD58_ + *k*_C96_)

^b^averaged CO_2_ water-air fluxes for the four parameterizations proposed (*k*_C96_, *k*_RC01_, *k*_B04_ and *k*_A09_)

^c^surface area of 265 km^2^ computed as the definite integral of bidimensional masks defined from latitude, longitude, and coastline information, using the ferret software.

**Figure 6:**
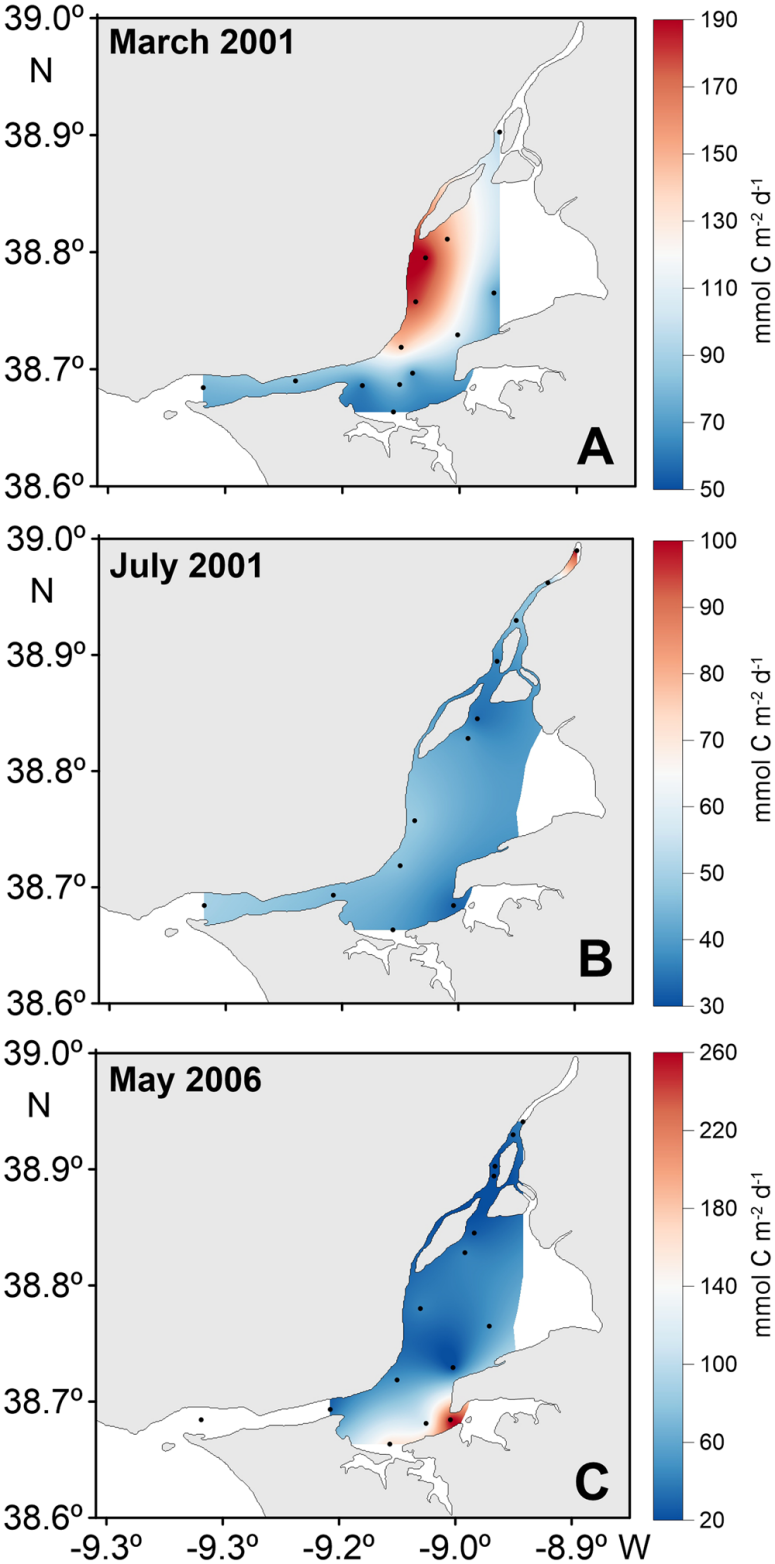
Water-air CO_2_ fluxes (mmol C m^−2^ d^−1^) distribution along Tagus estuary in (**A**) March 2001, (**B**) July 2001 and (**C**) May 2006. Figure generated using the software Surfer Version 12.8.1009 (http://www.goldensoftware.com/products/surfer), a surface mapping system from Golden Software, LLC. Coastline and bathymetry were created based on Google “Map data: Google, Earth” (https://www.google.pt/maps/) and on data SIO, NOAA, U.S. Navy, NGA, GEBCO version 20141103 (http://www.gebco.net).

When applying a sensitive analysis to the fluxes calculated for each of the various parameterizations, differences emerged varying from 8 to 30%. Hence, in order to minimize any substantial errors in the fluxes estimated due to the use of a single generic relationship, CO_2_ fluxes were averaged for the proposed parameterizations (*k*_C96_, *k*_RC01_, *k*_B04_ and *k*_A09_). A seasonal pattern of CO_2_ fluxes to the atmosphere was revealed in Tagus estuary, values increasing from 80.4 ± 87.7 mmol C m^−2^ d^−1^ in spring to 133.9 ± 89.3 mmol C m^−2^ d^−1^ in autumn (Table 2). Concerning spatial variability, the amplitude of fluxes variability was ~250 mmol C m^−2^ d^−1^ (calculated as the average of the difference between the least and greatest values observed for each sampling period) for the 10 surveys. Examples of CO_2_ fluxes distribution along the estuary in different year seasons (March 2001, July 2001 and May 2006) are shown in Fig. 6.

Overall, Tagus estuary functions as a source of CO_2_ to the atmosphere, being estimated an average annual flux of 33.6 ± 29.7 mol C m^−2^ yr^−1^ ^6^ and a total CO_2_ emission of 0.11 Tg C yr^−1^.

### Inorganic carbon balance

The following inorganic carbon fluxes were considered to establish the inorganic carbon balance in Tagus estuary: the riverine DIC input to the estuary, the estuarine DIC output to the adjacent coastal waters and the water-air CO_2_ flux. Mixing curves of DIC for each sampling period (exception of September 1999 and May 2007) were well reproduced by polynomial equations (Table 3). DIC fluxes and advective export of DIC added during estuarine transport (Internal DIC Flux; Table 3) were estimated^10^, and the magnitudes and temporal variation of inorganic carbon fluxes examined.

**Table 3:** Dissolved Inorganic Carbon (DIC) distributions and fluxes in Tagus estuary. Equations are polynomial equations used to fit the data from DIC *versus* salinity for each sampling transects. All equations have *p* < 0.05. C_0_ and C_S_ are, respectively, freshwater and seawater DIC concentrations. The flux of freshwater DIC is defined as (Q · C_0_), whereas the flux of internal DIC is (Q · (C_S_ − C_0_)) and the estuarine flux is (Q · C_S_). Q represents the Tagus River flow.

Season	Sampling dates	DIC (µmol kg^−1^) as a function of S	r^2^	C_0_^a^ (µmol kg^−1^)	C_S_^b^ (µmol kg^−1^)	Freshwater DIC Flux (10^6^ mol C d^−1^)	Internal DIC Flux (10^6^ mol C d^−1^)	Estuarine DIC Flux (10^6^ mol C d^−1^)
Spring	May 2000	43.1 S + 1540.7	0.774	1540.7	1540.7	51.0	0	51.0
Winter	March 2001	−29.2 S^2^ + 230.0 S + 1110.5	0.628	1110.5	2151.2	179.2	167.9	347.1
Summer	July 2001	23.5 · S + 1815.0	0.903	1815.0	1815.0	16.6	0	16.6
Summer	June 2002	−1.5 S^2^ + 63.4 S + 2481.5	0.527	2481.5	4243.2	17.8	12.6	30.5
Spring	May 2003	14.4 S + 1818.4	0.948	1818.4	1818.4	37.6	0	37.6
Winter	February 2004	0.7 S^2^ + 6.7 · S + 1832.7	0.602	1832.7	1445.8	42.5	−9.0	33.5
Spring	May 2006	−1.3 · S^2^ + 59.6 S + 1704.1	0.629	1704.1	3018.7	18.9	14.6	33.5
Autumn	November 2006	21.2 S + 2010.5	0.739	2010.5	2010.5	136.4	0	136.4
Annual mean values (Tg C yr^−1^)						0.27	0.10	0.37

^a^DIC concentration where the polynomial equation intersects the y-intercept (or the concentration at zero salinity)

^b^DIC concentration where the tangent at the marine end-member crosses the y-intercept

Freshwater end-member DIC concentration (C_0_; Table 3) did not vary much during all survey periods (1786.9 ± 155.8 µmol kg^−1^), except during an exceptionally intense river discharge in March 2001, and in June 2002, with low river flow and relatively high photosynthetic activity (Table 1). However, the estimates show that DIC riverine exports to the estuary varied from 16.6 × 10^6^ mol C d^−1^ (summer 2001) to 179.2 × 10^6^ mol C d^−1^ (winter 2001) (Table 3), with a mean annual flux of 0.27 Tg C yr^−1^.

It was observed that during the estuarine transport, generated DIC (C_S_ − C_0_) was higher in winter 2001 and lower (sometimes zero) in the other seasons. These internal fluxes ranged from 0 to 167.9 × 10^6^ mol C d^−1^, being estimated a mean annual internal flux of 0.10 Tg C yr^−1^ (Table 3). Fluxes from the estuary to the adjacent coastal waters varied from 16.6 × 10^6^ mol C d^−1^ (in summer 2001) to 347.1 × 10^6^ mol C d^−1^ (in winter 2001), corresponding to an annual flux of 0.37 Tg C yr^−1^ (Table 3). Thus, 27% of DIC was generated within the estuary, while the exported DIC to the atmosphere was 0.11 Tg C yr^−1^, which accounted for approx. 23% of the total DIC exported from the estuary.

## Discussion

**Figure 7:**
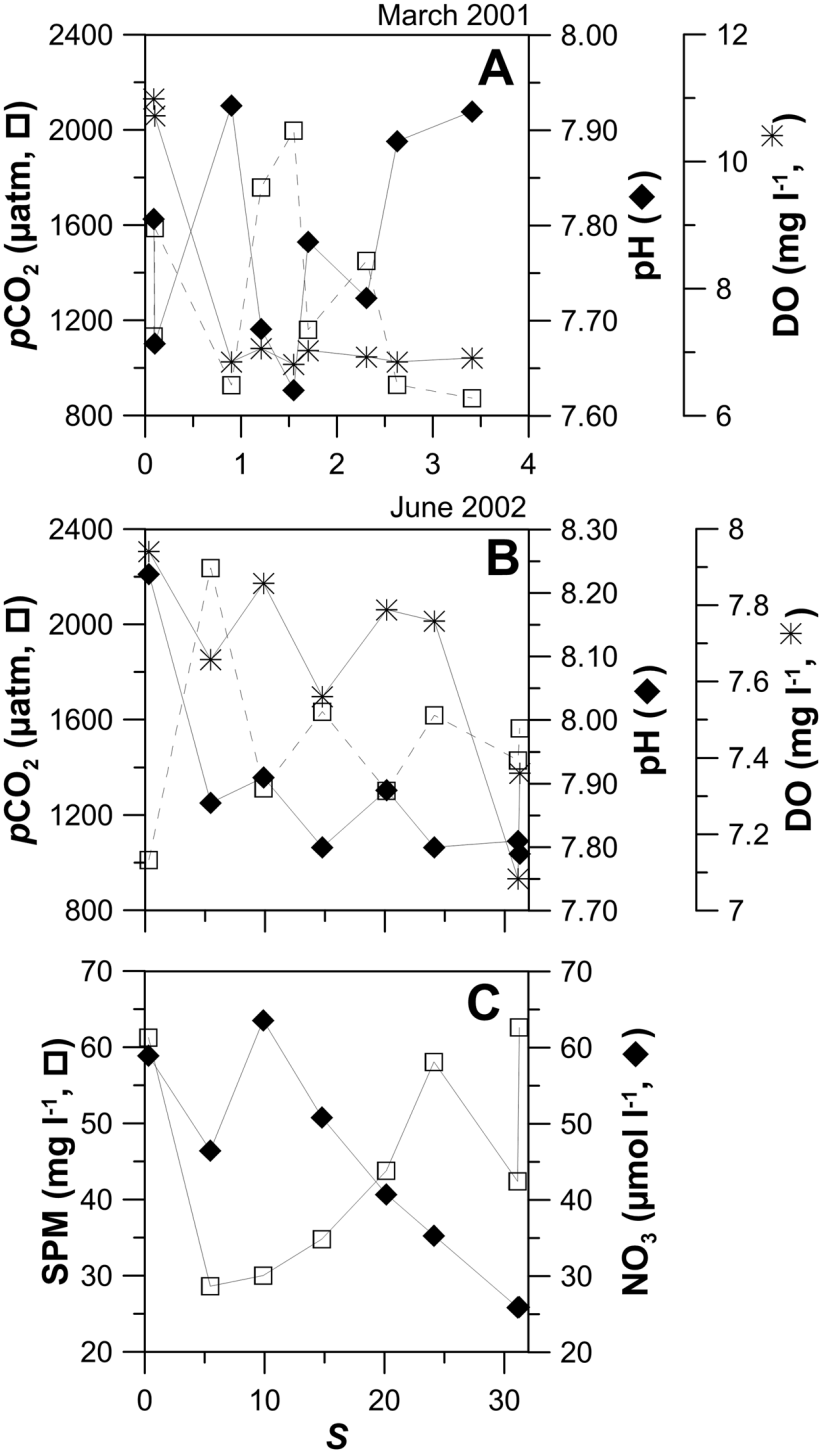
Distributions of CO_2_ partial pressure (*p*CO_2_), pH and dissolved oxygen (DO) along the salinity gradient in (**A**) March 2001 and in (**B**) June 2002. Also, distributions of (**C**) suspended particulate matter (SPM) and nitrate (NO_3_) in June 2002 along the salinity gradient in Tagus estuary.

Tagus waters environmental properties were strongly affected by the river discharge and showed a seasonal variability typical of a mid-latitude system, with most parameter values increasing from winter to spring/summer. Generally, river water carried higher contents of particulate material (Fig. 2D), nutrients (Fig. 7C) and phytoplankton (pers. comm.), than estuarine water. A tendency of a decrease ocean-ward was observed in winter, although high levels of SPM were present during the productive period in the 20–30 salinity range. This predominance of estuarine suspensions in the central/lower estuary can be interpreted as the result of salt marsh particles mixing and/or water circulation in the southern estuary, rather than the occurrence of biogeochemical processes. SPM values are comparable to values recorded for the Scheldt and the Thames estuaries and much lower (~14 times) than the ones in the highly turbid Gironde^11^.

pH values in the marine section were rather constant due to the seawater buffering capacity, while the riverine end-member pH varied largely being such variability attributed to runoff coupled to changes in biological activity. pH seasonal trends can be attributed to more runoff in winter and more intense primary productivity, in particular in spring/summer, upstream. Overall, pH values were higher in spring/summer and lower in autumn/winter (Fig. 3D).

TA and DIC riverine values were similar to those reported for the uppermost sections of other estuaries^12–15^. To a certain extent, the seasonal variability of TA and DIC was related to the river discharge. Highest values occurred during the low flow period when higher salinity water dominates the estuary and the lowest during intense river discharges. TA and DIC plots against salinity (Fig. 5B,C) show values increasing downstream. These trends have also been recorded in other estuaries^13, 14, 16–19^. The nearly-conservative behaviour can be explained by the counterbalance between sources and consumption of TA and DIC along the salinity gradient. Nevertheless, non-conservative relationships were observed in March 2001 (Fig. 5E,I), June 2002 (Fig. 5F,J), February 2004 (Fig. 5G,K) and May 2006 (Fig. 5H,L).

Tagus estuary was dominated by supersaturated CO_2_ conditions. Observed riverine *p*CO_2_ values (up to 9160 µatm) are similar to other estuaries^3, 17, 19–23^. The upper Scheldt, for example, revealed values of *p*CO_2_ as high as 9400 µatm^5^ and as 15500 µatm^3^. Similar high values are reported for the Saja-Besaya estuary^22^ (9728 µatm) at salinities below 5, and a range of 1000 to >6000 µatm at low-salinity areas in the Satilla and Altamaha Rivers^17^. As suggested by some authors^19, 21, 23–25^, high *p*CO_2_ values at riverine waters might be due to organic carbon mineralization in soils, river waters and sediments. Differences in inorganic carbon variability patterns in the two end-members indicate, beside the dilution effect, the complexity of interactions between input sources and processes acting along the estuary. Spatial trends and range of values for this estuary are also similar to those reported for other estuaries^4, 5, 14, 18, 19^.

Mixing curves are a commonly used approach for the interpretation of source/sink dynamics of estuarine constituents^10, 13, 26^. Assuming salinity as a satisfactory mixing indicator, the profiles of water properties as functions of salinity should be linear, if only mixing processes occur in an estuary. Thus, analysis of correlations between different parameters was used for identification of other processes in Tagus estuary.

As mentioned above, CO_2_ parameters behaved conservatively in most of the sampling periods, with the system dominated by mixing processes. But comparing the TA/DIC salinity profiles with theoretical mixing lines drawn between the two end-members, TA/DIC anomalies came out in the upper estuary at salinities below 10. In June 2002 an anomaly occurred at higher salinities in the middle estuary. Positive anomalies in TA/DIC mixing curves (TA/DIC concentrations lie above the theoretical mixing line) occurred in March 2001, as well in June 2002 and May 2006, and a negative anomaly occurred in February 2004. Thus, the non-linearity of TA and DIC distributions indicates that other processes are responsible for the inorganic carbon variability in some occasions. Actually, a non-conservative behaviour of TA and DIC in upper estuaries, mostly at salinities below 5, has been referred by some authors^27^.

In March 2001, an exceptional river discharge (1861 m^3^ s^−1^; Table 1) occurred, probably not allowing enough time for biogeochemical reactions to occur in the estuary^28^. However, a mechanism involving increase of *p*CO_2_ and decrease of pH and DO (Fig. 7A) has been hypothesized to be the aerobic respiration. Such mechanism would lead, nevertheless, to DIC increase and would have practically no effect on TA. Still, the simultaneous increase in both parameters (TA and DIC) was observed, pointing to a distinct process responsible for TA production, possibly calcium carbonate (CaCO_3_) dissolution. To assess this possibility, CaCO_3_ saturation state was calculated for calcite and aragonite (respectively Ω_c_ and Ω_a_), using the thermodynamic solubility products^29^. Calculations have shown that for this high flow period, the estuary was undersaturated with values of Ω_c_ and Ω_a_ varying from 0.01 to 1.02, eventually indicating CaCO_3_ dissolution. This process has referred to as the generator of alkalinity in Loire and Godavari estuaries^27, 30^.

In June 2002, a TA/DIC deviation from linearity was noticed in the central part of the estuary, at salinities approximately 20–30 (Fig. 5F,J). A production of TA of ca. 540 and of ca. 565 µmol kg^−1^ of DIC was estimated by comparing the theoretical mixing line to *in situ* values. The anthropogenic influence of Trancão River (Fig. 1) in that zone, with high loads of organic carbon and associated mineralization, may explain such increase in TA. Additionally, the concomitant decrease of pH and DO and increases of *p*CO_2_ (Fig. 7C), as well as of SPM and nitrate, decrease at that respective range of salinities (Fig. 7B), supports the occurrence of organic carbon mineralization. The considerable amount of suspended matter attained in that region (Fig. 7C) may have favoured some carbonate dissolution as well. In fact, it has been reported the occurrence of dissolution of CaCO_3_ and consequent generation of alkalinity in other estuaries, in zones of maximum turbidity, namely in Loire^27^ and in the highly turbid Gironde^31^ and Ems^32^.

A strong decrease of TA/DIC (430 and 640 µmol kg^−1^, respectively for TA and DIC) was observed during February 2004 (Fig. 5G,K) in the extremely low range of salinity (from 0 to 1), accompanied by a relatively conservative (r^2^ = 0.765, *p* < 0.05, n = 12) behaviour downstream. Simultaneously, *p*CO_2_ attained extremely high values (9160 µatm), contrasting with the values obtained in other sampling surveys (hardly attaining 4500 µatm; Table 1). This might be explained by the efflux of CO_2_ to the atmosphere in the very low salinities region, leading to the consumption of TA/DIC. Moreover, Chl *a* values (just up to 2.6 mg m^−3^; Table 1) reflecting winter conditions, support the above conclusion, since photosynthetic carbon fixation very unlikely would justify TA/DIC consumption. At salinity 20 another decrease of TA/DIC (~270 µmol kg^−1^) occurred, which also might correspond to CO_2_ degassing reflected in the decrease of *p*CO_2_ from 920 µatm to 670 µatm.

In May 2006, TA/DIC increase at low salinities seemed to be due to a combination of processes. Even if considerable primary productivity was underway in that region, reflected in elevated Chl *a* values (up to 73.4 mg m^−3^; Table 1), low *p*CO_2_ (487–650 µatm) and as well as relatively high pH (8.25; Table 1), consumption of TA/DIC did not occur, as expected for high phytoplankton biomass development. As no clear evidence of particulate organic matter mineralization was revealed, a possible explanation for production of DIC in that range of salinity could be mineralization of labile organic material (mainly glicids) produced by phytoplankton exudation and/or lysis. Thus, it means that photosynthetic activity might indirectly contribute to DIC increase, counteracting the expected decrease under conditions of relatively high productivity. Besides, another mechanism likely acting and leading to generation of alkalinity may have been dissolution of CaCO_3_, as by this period only slightly supersaturated conditions were present in the estuary.

It should be taken into account that the patterns of TA and DIC could also be related to other processes. As about one third of Tagus estuary surface is composed by intertidal areas, sediments are sites of organic matter degradation^33^. Moreover, salt marshes sediments store carbon and their pore waters enriched in DIC are transferred to the estuary waters by tidal pumping, also producing DIC. This mechanism has been reported to act in mangrove creek^34, 35^ as well. In addition, denitrification occurs in Tagus^36^, and it is known that such processes were responsible for TA increases in several estuaries^17, 31, 34, 37^.

The predominance of CO_2_ supersaturation conditions in Tagus estuary suggests that the system is dominated by heterotrophy^6^, which is backed by the O_2_ data (pers. comm.). The highest *p*CO_2_ values were noticed at the salinity ranges 0–5 and also 20–30 (Fig. 5D). Upstream *p*CO_2_ was most likely originated from riverine waters probably having no time to degas. *p*CO_2_ increase observed at higher salinities might be attributed to lateral transport of CO_2_ from salt marshes mainly located at the southern margin of Tagus in the north margin near the polluted Trancão River.

Studies carried out in Tagus marshes in 2001/2002 revealed CO_2_ supersaturation, and *p*CO_2_ values varying from 877 to 3950 µatm (Oliveira, unpublished data). Besides, other studies suggest that in tidally flooded salt marshes of some estuaries of Georgia/U.S.A, the CO_2_ supersaturation was controlled by inputs from organic carbon respiration^17, 38^. Also, CO_2_ supersaturation of three Georgia estuaries were due to CO_2_ inputs from the intertidal marshes and the rivers^39^. Thus, within Tagus estuary, mechanisms such as mixing of supersaturated freshwater with seawater, CO_2_ efflux to the atmosphere and carbonate dissolution seem to emerge as the major regulators of *p*CO_2_ spatial distribution.

As mentioned in another study^6^, higher CO_2_ emissions were generally related to more intense wind speed, although no statistical significance was observed (*p *> 0.05). Tagus estuary water current was found to largely affect the gas transfer velocity over it (~48% on average) (Table 2), an effect estimated by applying the ratio *k*_OD58_/(*k*_OD58_ + *k*_C96_).

The value is rather high when compared with estimates for other European estuaries, such as the Scheldt (25%)^40^ and Guadalquivir (30%)^41^. Assuming that water current and wind speed have an additive effect on *k* ^7^, the water current expression proposed by O’Connor and Dobbins^9^ was combined with the algorithms from Carini *et al*.^42^ and Raymond and Cole^43^. The outcome of the respective additive effect resulted in *k*’_C96_ = *k*_C96_ + *k*_OD58_ and *k*’_RC01_ = *k*_RC01_ + *k*_OD58_. Also, the possibility of a fetch effect on *k* has been investigated. Since the suspended matter levels present in the estuary were low to moderate (<165 mg l^−1^; Table 1) it was assumed that turbidity might have a rather limited effect on *k*.

Tagus estuary was always CO_2_ supersaturated inducing effluxes to the atmosphere at an average flux of 33.6 ± 29.7 mol C m^−2^ yr^−1^ leading to an average CO_2_ emission of 0.11 Tg C yr^−1^. Tagus CO_2_ fluxes are similar to the ones estimated for some inner estuaries (32.1 mol C m^−2^ yr^−1^) and analogous to fluxes of other European estuaries like Sado/Portugal (31.1 mol C m^−2^ yr^−1^), Gironde/France (30.8 mol C m^−2^ yr^−1^) and Guadalquivir/Spain (31.3 mol C m^−2^ yr^−1^)^4, 6^.

The nearly-conservative DIC distribution observed most of the time along the estuary implies that inputs and outputs are in equilibrium, meaning that the CO_2_ flux to the atmosphere must be balanced by net CO_2_ production in the water column or sediment. In fact, several researchers^13, 20, 38, 44^ referred the dynamic coupling between CO_2_ generation and evasion being extremely fast in estuaries.

Freshwater end-member DIC concentration did not vary much during all survey periods. Such near stable values suggest that the concentration of the inorganic carbon entering the estuary is, to a certain extent, independent of the season of the year and very likely independent of the Tagus discharges.

The relative contribution of riverine CO_2_ to the overall CO_2_ emission by the estuary (estimated after^45^) was ~6% in spring and ~10% in autumn. In fact, these values are very close to those reported for the very productive Scheldt estuary (10%)^45^ and for 11 European estuaries (median value 10%)^46^. Still, that proportion can be highly variable from one estuary to the other, for example for the Randers Fjord the riverine contribution reached a value of 50%^46^, for Guadalquivir estuary 30%^41^ and for Rhine estuary ~300%^46^. Hence, most of the emission of CO_2_ from Tagus estuary (~90%) was attributed to heterotrophic activity.

Our study suggests that changes in the Tagus estuary carbonate parameters seemed to be consequence of rather more complex feature than just the result of simple mixing of freshwater and seawater. Riverine/terrestrial runoff and occurrence of specific biogeochemical processes as photosynthesis, aerobic respiration, organic matter mineralization (degradation) and CaCO_3_ precipitation/dissolution might be responsible for carbonate variability. Probably, the pore waters rich in DIC transferred to the estuary waters by tidal pumping may also contribute to the occurred changes. The inorganic carbon budget revealed that 0.27 Tg C enter into Tagus estuary annually, while 0.37 Tg C are exported to the adjacent coastal waters. Overall, 23% of the total DIC exported from Tagus estuary is emitted to the atmosphere, and about 8% of riverine DIC is ventilated. Most of the estuarine emissions (average flux of 33.6 ± 29.7 mol C C m^−2^ yr^−1^) were attributed to heterotrophy.

## Methods

### Geographic coverage

Tagus Estuary (Fig. 1) is located at the southwest Portugal (38°36′–39°N, 08°54′–09°24′W) and supports important human communities and natural resources. It is an inundated valley with a submerged area of 320 km^2^ where ~40% are intertidal areas (~20 km^2^ salt marsh vegetation and ~80 km^2^ mudflats). This mesotidal estuary (tidal range 1–4 m) has a narrow and fault-controlled inlet channel separating two distinct regions: an outer wave-dominated area from an inner broad and tide-dominated part. Winds predominate from south and southwest during winter, rotating progressively to the northwest and north directions during spring, and maintaining these directions throughout the summer months. There is a strong seasonal hydrodynamic and biogeochemical variability due to seasonal fluctuations in meteorological conditions and river discharges (mean annual flow of 350 m^3^ s^−1^). There is also a strong horizontal gradient inside the estuary as a result of the hydrodynamic conditions, mostly tidally-controlled, with a dominant semidiurnal period and maximum amplitude of 4.8 m in spring tide. Middle estuarine areas, and to a lesser extent upper areas, have more stable and homogenous conditions, displaying a higher residence time. Lower estuarine areas, influenced by the tidal regime, are characterized by high variability. The mean residence time of water in the estuary varies between 26 and 8 days^47^. The system is vertically well-mixed and has a mean tidal prism of 600 × 10^6^ m^3^, about a third of the mean volume.

Even with recent reduction in organic loadings due to the implementation of considerable sewage treatment plants, the Tagus estuary is under intense anthropogenic disturbance, with a major population centre within its catchment basin (~2.3 million of inhabitants).

### Sampling, analysis and calculation methods

The sampling period was from 1999 to 2007, covering an area extending from the estuary mouth to the point where freshwater was encountered. Sampling locations were selected to provide a full coverage of the salinity gradient (from 0 to 35). Surface seawater samples were collected at ebb conditions with Niskin bottles, for a total of 18 sites along a ~50 km stretch of the estuary (Fig. 1). Temperature (*T*) and salinity (*S*) parameters were determined *in situ* with a CTD (Conductivity - Temperature - Depth) Aanderaa probe. Salinity was calibrated with an AutoSal salinometer using IAPSO standard seawater, with a variation coefficient of 0.003%.

Dissolved oxygen (DO) was analysed following the Winkler method^48^ using a whole-bottle manual titration. The coefficient of variation associated with the method ranged from 0.08 to 0.25%. pH was measured immediately after sample collection at 25 °C, using a Metrohm 704 pH-meter and a combination electrode (Metrohm) standardised against 2-amino-2-hydroxymethyl-1,3-propanediol seawater buffer (ionic strength of 0.7 M), at a precision of 0.005 pH units^49^. Total alkalinity (TA) samples were filtered through Whatman GF/F (0.7 μm) filters, fixed with HgCl_2_ and stored (refrigerated not frozen) until use. Samples were then titrated automatically with HCl (~0.25 M HCl in a solution of 0.45 M NaCl) past the endpoint of 4.5^49^, with an accuracy of ±2 μmol kg^−1^. The respectively accuracy was controlled against certified reference material supplied by A.G. Dickson (Scripps Institution of Oceanography, San Diego, USA).

Chlorophyll *a* (Chl *a*) was determined by filtering triplicate aliquots of ~150 ml water through Whatman GF/F filters (0.7 µm) under a 0.2 atm vacuum, and immediately frozen and later extracted in 90% acetone for analysis in a fluorometer Hitachi F-7000, calibrated with commercial solutions of Chl *a* (Sigma Chemical Co.). The variation coefficient was 1.8%. For suspended particulate matter (SPM) determinations, six aliquots of 100–1000 ml water samples were filtered through pre-combusted (2 h at 450 °C) Wathman GF/F filters and determined gravimetrically (drying at 70 °C). The respective filters were subsequently used for particulate organic (POC) and inorganic (PIC) carbon determinations using a CHN Fissons NA 1500 Analyser, with acetanilide as the calibration standard. System blanks were obtained by running several empty ashed tin capsules. The analyser provides a measure of total carbon, so the inorganic fraction was removed by drying the filters at 450 °C. The method precision was of 0.47%.

### Meteorological data

Wind speed and direction were measured *in situ* with a Vaisala^®^ meteorological station (Datalogger Campbell Scientific CR510) coupled with a MetOne 034 A anemometer. Continuous measurements were acquired with 1-minute intervals at 11 m height. Wind speed was referenced to a height of 10 m (u_10_)^50^. We assume one standard deviation of ± 2 m s^−1^ as wind speed error. Atmospheric CO_2_ data was obtained from the Terceira Island’s reference station (Azores, Portugal, 38.77°N 27.38°W), from the network of the National Oceanic and Atmospheric Administration (NOAA)/Climate Monitoring and Diagnostics Laboratory (CMDL)/Carbon Cycle Greenhouse Gases Group (CCGG)^51^. Some algorithms^49^ were used to convert observed atmospheric CO_2_ content in mole fraction (in dry air) to wet air values. Direct measurements of atmospheric CO_2_ partial pressure made on-board was only available for some sampling periods. Significant correlations were found out between Terceira data and shipboard data (r^2^ = 0.910, *p* < 0.05, n = 45) and the discrepancies lied just between 3 and 13 µatm. The impact of using Terceira data on this study was considered negligible.

### Estimated parameters

pH values corrected to *in situ* temperature were calculated from total alkalinity (TA) and *in situ* pH and temperature^52^. For these calculations the carbon dioxide constants of Millero *et al*.^53^ were applied.

The partial pressure of CO_2_ in seawater (*p*CO_2_) and the dissolved inorganic carbon (DIC) were calculated from the *in situ* temperature, TA and corrected pH, using the carbonic acid dissociation constants given by Millero *et al*.^53^ and the CO_2_ solubility coefficient of Weiss^54^. Errors associated with *p*CO_2_ and DIC calculations were estimated to be ± 10 μatm and ± 5 μmol kg^−1^, respectively (accumulated errors on TA and pH).

The water-air CO_2_ fluxes (CO_2_ Flux) were computed according to:

1 $${{\rm{C}}{\rm{O}}}_{2}\,{\rm{F}}{\rm{l}}{\rm{u}}{\rm{x}}=k\cdot {{\rm{K}}}_{0}\cdot {\rm{\bigtriangleup }}p{{\rm{C}}{\rm{O}}}_{2}$$

where *k* is the CO_2_ gas transfer velocity, K_0_ the solubility coefficient of CO_2_ and Δ*p*CO_2_ the water-air gradient of *p*CO_2_. Positive CO_2_ flux indicates emission of CO_2_ from water to the atmosphere and negative flux the opposite direction.

Some authors^7, 55, 56^ suggest that *k*–wind speed relationships are site-specific. More recently, it was argued^8^ that wind, water current, surface area and turbidity, all significantly affect *k* in estuaries. Various measurement techniques on the gas transfer velocity in estuaries have been implemented, using methods such as dual tracer addition^42^, natural gas tracer^57^ or floating dome technique^7^. The tracer methods involve long term measurement of *k* over the entire estuary, while the floating dome technique is a short term measurement affected by the system heterogeneity that is typical in estuaries. In any case, the selection of a particular value for *k* will affect the overall representation of the net ecosystem metabolism. Since *k* was not determined *in situ* in Tagus estuary, we based our calculations to bracket the most likely value for *k* on parameterizations used on similar studies: (1) from a SF6 release experiment in the Parker River and estuary^42^ (hereafter referred to as C96), (2) from a compilation of published *k* values in various rivers and estuaries, using different methodologies^43^ (hereafter referred to as RC01), (3) considering the contribution of the water current^7, 9^ (hereafter referred to as B04), and (4) based on a generic equation that gives *k* as a function of water current velocity, wind speed, estuarine surface area and suspended matter content^8^ (hereafter referred to as A09). The choice of these formulations was motivated by the relative similarity between the Tagus estuary and the systems studied by the mentioned studies, in their physical characteristics (e.g., shallow, well-mixed, influenced by tides). Water current and tidal height data at Tagus estuary were obtained with hindcast simulations using the MOHID Modelling System (www.mohid.com), based on real forcing for river discharge, tide and wind^58–60^.

### Estimated DIC fluxes

The internal flux of dissolved constituent was estimated based on mixing curves^10^, by quantifying how much DIC was added by net heterotrophy during estuarine transport through DIC *versus* salinity plots. Whenever the distribution of a dissolved constituent is continuous and predictable using simple polynomial equations, C_0_ is where the polynomial equation defining DIC concentrations intersects the y-intercept (or the concentration at zero salinity), and C_S_ is the concentration of the constituent where the tangent at the seawater end-member crosses the y-intercept. Still, total exported flux from the estuary is given by (Q · C_S_), where Q is the freshwater flow, (Q · (C_S_ − C_0_)) the internal flux, and (Q · C_0_) the flux from the freshwater end-member.

### Statistical analysis

Exploratory analysis and statistical procedures were implemented using the statistical software Statistica 6.0^®^ (Statsoft Inc., 2001). Differences between sampling periods in the measured/calculated physical-chemical and biological parameters, were assessed using an analysis of variance (ANOVA), and differences between means have been considered statistically significant for *p* < 0.05. The dominant processes influencing surface water chemistry were identified using linear correlations between the system parameters.
